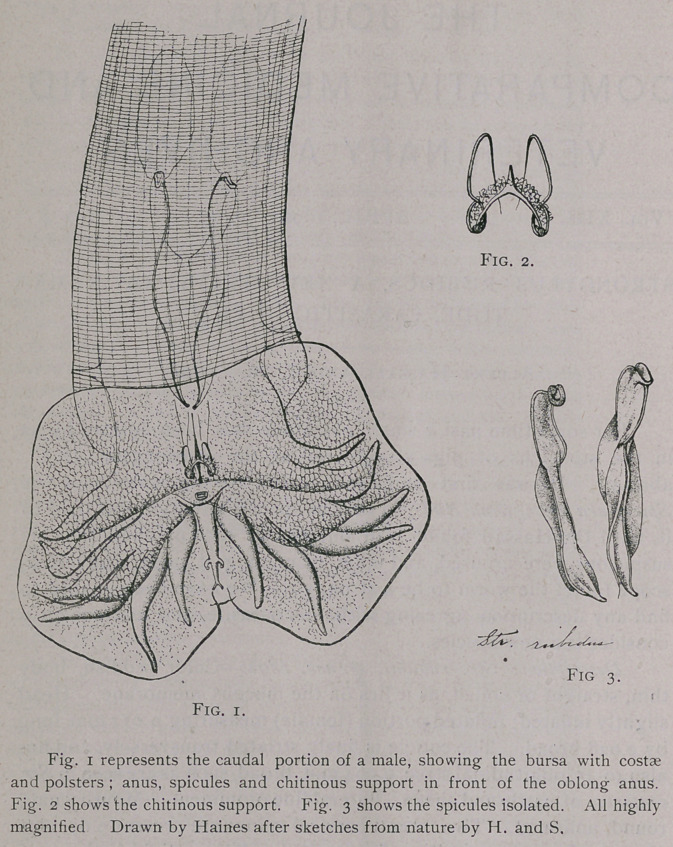# Strongylus Rubidus, a New Species of Nematode, Parasitic in Pigs

**Published:** 1892-04

**Authors:** Albert Hassall, C. W. Stiles

**Affiliations:** B. A. I., U. S. Dep’t of Agriculture; B. A. I., U. S. Dep’t of Agriculture


### THE JOURNAL
OF
### COMPARATIVE MEDICINE AND VETERINARY ARCHIVES.

VOL. XIII. APRIL, 1892. No. 4.

### STRONGYLUS RUBIDUS, A NEW SPECIES OF NEMATODE, PARASITIC IN PIGS.

ALBERT HASSALL AND C. W. STILES.

For some time past a small nematode has been noticed by us in the stomachs of pigs slaughtered at the Washington, D. C., abattoir. It was first supposed to be young specimens of *Spiroptera strongylina Rud.*, so that no special attention was paid to it, until Dr. Hassall found it in one pig in such numbers that his suspicions were aroused, and upon examining it under the microscope found the worm to be a strongyle. We have been unable to find any description agreeing with this parasite, and consequently consider it a new species.

*Description:* *Str. rubidus, sp. nov. 1892.*—Color reddish, body thin, straight or coiled, as it lies on the mucous membrane Head slightly inflated; inflated portion (female) measuring 0.012 mm. long by 0.028 broad. The cuticle is finely striated transversely, and has also 40-45 longitudinal striæ, 0.004 mm. broad, such as are seen in *Str. contortus* of cattle and sheep. Lateral line prominent. Mouth small, round, unarmed. Two lateral cervical spines are present 0.67 mm. (female) from the mouth. The cuticular lining divides the œsophagus indistinctly into two portions. The anterior portion is 0.24 mm. long and about 0.02 wide; the posterior portion is 0.4 mm. long and 0.042 wide at the posterior end. The muscular striation of the posterior portion is much broader than that of the anterior portion. The division between the two portions lies just before the ventral pore. The intestine is cylindrical, 0.04 in diameter, greyish-black, and winds spirally with the genital organs. Cephalic glands prominent, 0.99 long, 0.02 broad. Excretory pore 0.23–0.29 from the mouth (female).

FIG. 1.

FIG. 2.

FIG 3.

Fig. 1 represents the caudal portion of a male, showing the bursa with costæ and polsters; anus, spicules and chitinous support in front of the oblong anus. Fig. 2 shows the chitinous support. Fig. 3 shows the spicules isolated. All highly magnified Drawn by Haines after sketches from nature by H. and S.

*Male.*—5 mm. long, 0.087–0.128 broad. Bursa 0.3 broad by 0.195 long, two lateral lobes continuous anteriorly, distinct posteriorly and connected by a small median lobe. Two transverse ridges divide the inner surface of the bursa into four polsters, two of which lie each side of the median line. Costæ (see fig. 1): 1-6 all separate for nearly their entire length. Middle lobe with two pairs of rays. Spicules double, 0.13 long, 0.02 broad (fig. 3). Anus square or oblong, 0.01 by 0.004. Anterior to the anus is a peculiar forked chitinous support (fig. 2) 0.012 broad by 0.014 long, the fork opening towards the anus; a narrow chitinous strip, 0.06 long, is seen dorsal of this fork, and probably corresponds to the unpaired chitinous support found in other strongyles, as *St. contortus*, although it is very much more simple in structure.

*Female.*—8.–8 5 mm. long by 0.11 broad; anus 0.68 from tip of tail. Vulva 1.3–1.5 mm. in front of the anus. Directly caudad of the vulva is a small semi-lunar cuticular fold, about 0.04 long by 0.013 broad. Vagina is bottle-shaped and at right angles to the body wall, 0.056 long · two uteri are present, branching off at right angles to the vagina. The first 0.2 of the uterus has a very prominent cuticle lying in folds, and is surrounded by a thick striated tissue, resembling muscle. Eggs 0.045 by 0.036; clearage begins in the mother.

So far as it is possible for us to state at present, the worm appears to have scarcely any clinical importance. It has been found in some cases in such immense numbers that the mucus of the stomach seemed to be blood-stained, while at other times but few were present. A number of cases were found in which there was an extensive ulceration in the stomach, but this was not constant in pigs containing the parasites; in fact some ulcers were found in stomachs where we could discover neither *Str. rubidus* nor *Sp. strongylina*, so that we do not at present feel justified in considering the worms as the cause of ulceration, although we suspected it for some time. It can, however, be stated that in all cases where large numbers of *St. rubidus* were found, there was an excess of thick mucus present, which gave us the impression that the catarrhal state was due to irritation by the worms.

In some lots of hogs we examined *St. rubidus* was present in 75 per cent., while in others the percentage ran as low as 25. As the parasite is extremely small, it is often overlooked, and this may account in some degree for the low percentage sometimes found. It may be added that we have not counted in the 75 per cent. and 25 per cent. those cases where only one or two worms were found, for these could easily have been transferred to the stomachs by our hands in examination, or by contact with other stomachs in the same box, for we examined them after they had been turned inside out in the process of preparing them for "hogshead cheese."

B. A. I., U. S. Dep't of Agriculture, Feb. 1, 1892.

## Figures and Tables

**Fig. 1. Fig. 2. Fig. 3. f1:**